# Unveiling peripheral neuropathy and cognitive dysfunction in diabetes: an observational and proof-of-concept study with video games and sensor-equipped insoles

**DOI:** 10.3389/fendo.2024.1310152

**Published:** 2024-03-01

**Authors:** Antao Ming, Elisabeth Lorek, Janina Wall, Tanja Schubert, Nils Ebert, Imke Galatzky, Anne-Katrin Baum, Wenzel Glanz, Sebastian Stober, Peter R. Mertens

**Affiliations:** ^1^ University Clinic for Nephrology and Hypertension, Diabetology and Endocrinology, Otto-von-Guericke University Magdeburg, Magdeburg, Germany; ^2^ University Clinic for Neurology, Otto-von-Guericke University Magdeburg, Magdeburg, Germany; ^3^ Institute of Cognitive Neurology and Dementia Research, Otto-von-Guericke University Magdeburg, Magdeburg, Germany; ^4^ Artificial Intelligence Lab, Otto-von-Guericke University Magdeburg, Magdeburg, Germany

**Keywords:** diabetes mellitus, cognitive dysfunction, peripheral neuropathy, sensor-equipped insoles, video games, machine learning

## Abstract

**Background:**

Proactive screening for cognitive dysfunction (CD) and peripheral neuropathy (PNP) in elderly patients with diabetes mellitus is essential for early intervention, yet clinical examination is time-consuming and prone to bias.

**Objective:**

We aimed to investigate PNP and CD in a diabetes cohort and explore the possibility of identifying key features linked with the respective conditions by machine learning algorithms applied to data sets obtained in playful games controlled by sensor-equipped insoles.

**Methods:**

In a cohort of patients diagnosed with diabetes (n=261) aged over 50 years PNP and CD were diagnosed based on complete physical examination (neuropathy symptom and disability scores, and Montreal Cognitive Assessment). In an observational and proof-of-concept study patients performed a 15 min lasting gaming session encompassing tutorials and four video games with 5,244 predefined features. The steering of video games was solely achieved by modulating plantar pressure values, which were measured by sensor-equipped insoles in real-time. Data sets were used to identify key features indicating game performance with correlation regarding CD and PNP findings. Thereby, machine learning models (e.g. gradient boosting and lasso and elastic-net regularized generalized linear models) were set up to distinguish patients in the different groups.

**Results:**

PNP was diagnosed in 59% (n=153), CD in 34% (n=89) of participants, and 23% (n=61) suffered from both conditions. Multivariable regression analyses suggested that PNP was positively associated with CD in patients with diabetes (adjusted odds ratio = 1.95; 95% confidence interval: 1.03-3.76; *P*=0.04). Predictive game features were identified that significantly correlated with CD (n=59), PNP (n=40), or both (n=59). These features allowed to set up classification models that were enriched by individual risk profiles (i.e. gender, age, weight, BMI, diabetes type, and diabetes duration). The obtained models yielded good predictive performance with the area under the receiver-operating-characteristic curves reaching 0.95 for CD without PNP, 0.83 for PNP without CD, and 0.84 for CD and PNP combined.

**Conclusions:**

The video game-based assessment was able to categorize patients with CD and/or PNP with high accuracy. Future studies with larger cohorts are needed to validate these results and potentially enhance the discriminative power of video games.

## Introduction

In patients with diabetes mellitus peripheral neuropathy (PNP) and cognitive dysfunction (CD) are significant and debilitating complications that may develop at the same time (1–3). Studies indicate that 25-50% of patients with diabetes manifest PNP, with severity scores correlating with advanced age, diabetes duration, and poor glycemic control (4–6). At the same time diabetes type 2 patients exhibit a 1.5 to 2.5-fold elevated risk of cognitive decline relative to a non-diabetes cohort. In patients diagnosed with diabetes type 1 an 80% augmented likelihood of cognitive decline exists (7, 8). PNP is a condition predisposing towards diabetic foot syndrome with complications such as skin ulcerations, infections. At its extreme this complication necessitates limb amputations (9). CD in patients with diabetes increases the risk of disability and falls, reduces quality of life, and leads to higher mortality rates (10). Cognitive decline in diabetes has been ascribed to conditions of chronic hyperglycemia with structural changes of the brain, such as brain atrophy, microvascular lesions, and activation of brain microglia resulting in demyelination (11–13). The aetiology of PNP involves microvascular damage patterns and inflammation of nerve structures (14–16). Beyond these issues, CD and PNP may have overarching commonalities and shared pathomechanisms (17, 18). Cohort studies have reported that patients with diabetic peripheral neuropathy (PNP) had worse cognition in the domains of memory and processing speed compared to patients without PNP (19). Cognitive dysfunction was associated with PNP, which was solely diagnosed by monofilament testing (20). However, the precise interrelationships and mechanisms underpinning CD and PNP in diabetes remain largely elusive, warranting further investigations (21, 22). It has to be emphasized that all subgroups of patients exist, with CD or PNP in isolation or with both conditions combined. Timely screening for CD and PNP should allow early interventions (2). These may include correction of vitamin B12 deficiency that often evolves with metformin medication. In addition, supplementation with iron is often needed to prevent deficiencies. PNP screenings in outpatient clinics typically commence with questionnaires on medical history and neuropathic symptoms, complemented by vibration and pressure sensation tests (pinprick), as advocated by the National Institute for Health and Care Excellence (5, 23). Advanced evaluations by nerve conduction studies (NCS) and skin biopsies are rarely executed given their invasive nature (24, 25). The common repertoire of caring physicians to detect CD by neuropsychological evaluation include the Mini-Mental State Examination (MMSE) or Montreal Cognitive Assessment (MoCA). There are several limitations in classifying cognition and dementia. One issue is timing, given that dementia may only be diagnosed when cognition is impaired for at least 6 months (26). Questionnaires are often complemented by brain imaging, such as MRI, and blood tests for pertinent markers, such as tau or amyloid proteins (10, 27). The clinical assessments of PNP and CD rely on patient feedback, which introduce subjectivity that can lead to intended or unconscious inaccuracies (24, 27). More precise and specialized tests (e.g. NCS, magnetic resonance imaging) outperform the former ones, yet their accessibility varies (28). Comprehensive screens, albeit crucial for high-risk cohorts, are regrettably not universally administered, with tests conducted in only a minor share of 35% of such populations (29). Constraints encompass healthcare infrastructure limitations, patient awareness deficits, and healthcare disparities, underscoring an exigent call for innovation in this field (30). The aforementioned high prevalence rates together with severe co-morbidities constitute a condition where diagnosis of CD and/or PNP beyond extensive clinical examination is desirable.

Computerized games for health diagnostics represents a transformative approach in the medical field (31). Potentially these games provide avenues for at home monitoring and rehabilitative interventions (31–33). Regarding CD, these tools may provide fine granularity in detecting aberrations across diverse cognitive domains, such as memory and spatial navigation (34). Their setup depends on patient compliance. Feasibility of home-based evaluations should foster physician-independent assessments (35, 36).

Proof-of-concept studies are critical in the early phases of clinical research, primarily to evaluate the feasibility and efficacy of new treatments (37). In this proof-of-concept study, we aimed to determine the existence and severity of CD and PNP in a diabetes cohort and to investigate the possibility of identifying the respective conditions using machine learning algorithms and key features extracted from playful games controlled by sensor-equipped insoles.

## Materials and methods

### Study design and participants

The development of the game platform and the clinical study were carried out at the Clinic for Nephrology and Hypertension, Diabetes and Endocrinology at the Otto-von-Guericke University Magdeburg, Germany. This study was conducted following endorsement by the local ethics committee (28/17 on 13.03.2017).

From 07/2020 to 07/2023 patients were screened and asked to provide written informed consent after a detailed explanation of the study protocol, test procedure, and data policy. Patients with diabetes mellitus aged above 50 years were eligible for the study, except for those with the following conditions: macroangiopathy of the lower extremities; physical deformities (amputations, foot and leg deformities requiring orthopedic shoe fitting); manifest neuropathic foot ulceration; visual disorders including visual acuity of less than 0.8 (except for corrected myopia and hyperopia); muscular diseases/motor diseases; myocardial infarction <12 weeks; advanced heart failure (NYHA III or IV); transient ischemic attacks; stroke; tremor; lack of ability to give consent for any reason; lack of ability to use a mobile phone. A visual field test by perimetry was not part of the entry examination of the patients into the study.

### Clinical evaluation and examinations for CD and PNP

An extensive questionnaire was administered, covering aspects of past medical history, specifics on diabetes mellitus manifestation (type, duration, therapeutic approach, sensory disturbances, movement constraints in daily life), symptoms of autonomic diabetic neuropathy (dizziness, cardiac arrhythmia, urinary irregularities, sweating), comorbidities related to diabetes, and daily activities (sports, handedness, foot dominance). All patients were carefully examined for PNP by a study physician. The German versions of the Neuropathy Disability Score (NDS) and Neuropathy Symptom Score (NSS) were employed for assessment (38). Both tools are frequently utilized in the clinical setting and cover neuropathic symptoms and signs. The NDS evaluates ankle reflexes, vibration perception, pin-prick, and temperature sensation (cold tuning fork) at the digitus I, with a maximum abnormal score of 10. Scores of 3-5 indicate mild, 6-8 moderate, and 9-10 severe neuropathic signs (39). The NSS is utilized to obtain information on pain and discomfort sensations in the legs and applies a scoring system that may also reach a maximum of 10 for the worst condition. Scores of 3-4 suggest mild, 5-6 moderate, and 7-10 severe symptoms (40). Diagnosis of PNP in our study necessitated either a score of NDS≥6 or a combination of NDS≥3 and NSS≥5 according to the clinical practice guidelines of the German Diabetes Association (38, 41, 42).

Cognitive proficiencies of the participants were evaluated using the Montreal Cognitive Assessment (MoCA) test that scores out of 30 points, with higher scores indicating better cognitive performance (43). One point was added to the MoCA score for persons with 12 or fewer years of education. A score below 26 was considered as CD (44).

### Gamified assessment for PNP and CD

**Figure 1:**
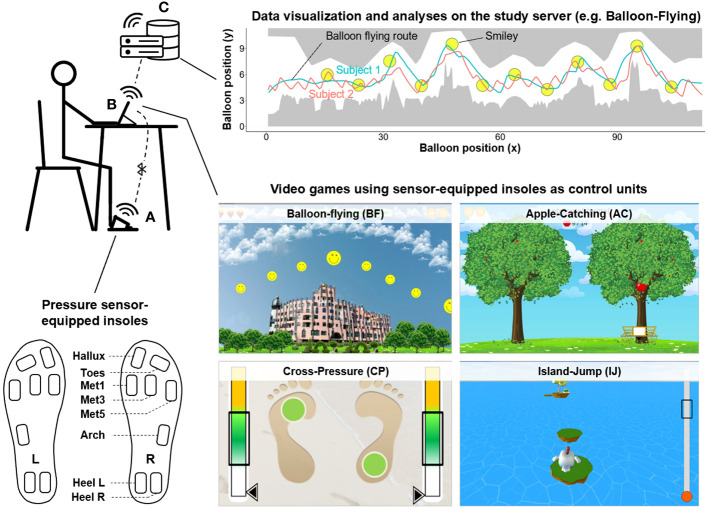
Game-based assessment of PNP and CD. Game-based assessment of PNP and CD. **(A)** Sensor-equipped gaming insoles with eight pressure sensors were linked to a tablet game application via Bluetooth. The setup allowed the participant to play games while sitting by modulating plantar pressure values. **(B)** Each gaming session included four games: Balloon-Flying (BF), Apple-Catch (AC), Cross-Pressure (CP), and Island-Jump (IJ). After playing, the data was uploaded to the study server as a data package. The participants received a short summary of their performance, such as scores, levels, and capabilities (from beginner to expert). **(C)** Physicians accessed visualized data to compare participant performance against maximum achievement levels. Utilizing the game-derived data from participants, pre-trained AI models extrapolated predictions related to PNP and CD, thereby supporting clinicians in decision-making for subsequent therapeutic interventions.

All participants underwent game sessions with size-matched shoes harboring sensor-equipped insoles (ActiSense System^®^, IEE S.A. Luxembourg, **Figure 1A**). Each insole was equipped with an Electronic Control Unit (ECU) and eight pressure sensors (5.57 cm^2^ HD002 force sensing resistors) strategically located in regions such as the heel, lateral arch, metatarsals 1, 3, and 5, hallux, and toes. These sensors measured plantar pressures with a sensitivity of 3.4 mbar ranging between 250 mbar and 7 bar and a maximum sampling rate of 500Hz.The ECU facilitated data synchronization with smart devices, automatic foot side detection, 16 GB internal storage, and a 10-hour energy supply. The sensors were covered with foil and were integrated into the footwear with a supportive ethylene-vinylacetat-30 layer and a protective sponge layer.

During gaming, plantar pressures were measured at 200 Hz and transmitted to the application via Bluetooth 5.0 that enabled seamless game control. Participants sat on an armrest-free chair and oriented towards an Android tablet (Samsung Galaxy Tab A T580) with the game application connected to insoles. Initial calibration was conducted via eight standardized procedures to acquire thresholds for pressure normalization into a range of 0-1 (**Supplementary Figure 1**). Participants then underwent standardized tutorials to acquaint themselves with the game controls and setup. The session encompassed four sequential games: Apple-Catch (AC), Balloon-Flying (BF), Cross-Pressure (CP), and Island-Jump (IJ), completed within 15 minutes (**Figure 1B**).

Overview on video game composition and challenges (**Figure 1B**): During the BF game, individuals maneuvered a balloon across a skyline, with its altitude dictated by the different pressure applied to the forefoot regions of the insoles. Players strived to circumnavigate obstacles and accumulate points, represented by smileys, necessitating swift corrective actions to avoid collisions and consequent game restarts. In the AC game, participants were immersed in an autumnal scenario where they utilized plantar pressure (sensors Met1/3/5) to operate a carriage, endeavoring to catch apples that fell at regular intervals from a tree. The necessary precision in sustaining the target pressure ensured optimal carriage positioning for a successful catch. Following each attempt, the carriage automatically returned to a central baseline. In the CP game, participants were directed to apply varying degrees of pressure denoted by green (low) and yellow (high) indicators on specific foot areas for designated durations. Achievements were affirmed through graphical indicators, and the game progressed to the subsequent task if no valid response was registered within 25 seconds. The IJ game engaged users in guiding a virtual bird across islands through plantar pressure applications, dictating both the jump distances and directions. In case that the pressure was not adjusted within the indicated range the virtual bird fell into the ocean and the attempt had to be repeated. The attainment of optimal scores required strict adherence to predefined pressure thresholds, with deviations prompting game restarts. More details about game design and feature extraction methods are provided in the **Supplementary Materials** (**Supplementary Figures 2–5**), as well as an introduction video on the game-based application. Collectively, these games engaged users in complex tasks necessitating nuanced control of foot pressure, potentially fostering assessment of cognitive function and motor/sensory nerve status through an engaging and interactive medium.

At the end of the gaming session, the collected data were analyzed by machine learning algorithms (see below) and results were visualized (**Figure 1C**). Participants received a spider chart diagram as feedback which highlighted key capabilities, such as skillfulness, reaction time, sensation, endurance, muscle strength, and balance.

### Game feature extraction

Feature extraction of distinct parameters from defined tasks and task combinations (TC) for the four games was conducted to assess the performance of study participants (**Supplementary Figure 6**). Utilizing the AC game as an example, its duration was standardized, encompassing a total of 14 tasks (apples to be caught). Specific parameters per task were delineated, serving as the foundation for the evaluation of player performance. These game parameters were identified as primary features, such as the reaction time observed in the initial task of the AC game. Furthermore, the concept of task combination (TC) was introduced, wherein a set of game tasks with analogous specifications were amalgamated. For instance, TCL1 encompassed all tasks that depended on the left foot to control carriage movement for apple collection. Conversely, the corresponding TCR1 comprised all tasks that relied on the right foot. The sum, mean, and standard deviation of primary features across game tasks of TCs were classified as secondary features. For instance, within the AC game, the reaction time of TCL1 constituted a secondary feature, calculated from the average reaction times across tasks involving the left foot. In summary, a total of 5,244 distinct parameters, reflecting the players’ performance across the four games, were extracted for each gaming dataset (per participant).

### Statistical analysis

Descriptive statistics are presented in proportions and frequencies for categorical variables. For continuous variables, mean (standard deviation [SD]) and median (interquartile range [IQR]) are used for the description of normally and non-normally distributed data, respectively. For comparison between groups, Chi-square tests were performed on categorical variables. The Shapiro-Wilk normality test was utilized to determine the normal distribution of continuous variables. For normally distributed variables, the group differences were computed with t-tests. Otherwise, Mann-Whitney U tests were performed on variables. In addition, the Kruskal-Wallis H test or one-way ANOVA was utilized for comparing multiple groups. Two-sided P values below 0.05 were considered as statistically significant. Pairwise tests were performed automatically among multiple groups with the correction of the P values using the Holm–Bonferroni method (45). Relationships between variables were determined through correlation tests and linear regression, using Pearson or Spearman correlation based on data distribution. Missing data was not included in the correlation analysis.

To analyze the association between CD and PNP, the cardinality matching method was initially employed to balance covariates (gender, age, weight, BMI, diabetes type and diabetes duration) between groups (i.e. no CD versus CD) (46). We performed logistic regression analyses to determine whether PNP is risk factor for CD in patients with diabetes. We initially performed a univariate regression analysis and thereafter included covariates such as age, gender, weight, BMI, type of diabetes, duration of diabetes according to clinical relevance and findings from previous research (20). In the models, clinical scores were evaluated as continuous variables and converted to categorized variables if possible with the first stage considered as a reference (e.g. NSS and NDS). Subcohort analyses based on gender and diabetes type were conducted as well. Study participants with missing MoCA data were excluded from the analyses. Data processing and analyses were performed using R programming language (version 4.2.1) and related open-source libraries (e.g. caret version: 6.0-94, glmnet version: 4.1-7, ggplot2 version: 3.4.2), along with Storm Statistical Platform (47, 48). A complete list of R packages is provided in the **Supplementary Materials**.

### Model development for CD and PNP classification

The main outcome of the proof-of-concept study was the correct assignment of the patients into the subcategories without CD/PNP or CD or PNP or both conditions CD/PNP combined. Three binary classification models were developed to discriminate between the following patient groups: [+PNP, -CD] versus [-PNP, -CD], [-PNP, +CD] versus [-PNP, -CD], and [+PNP, +CD] versus [-PNP, -CD] using game parameters extracted from acquired data sets, as well as six individual risk profiles. During the variable selection process, highly inter-correlated game parameters were excluded using a correlation coefficient of 0.5 (49). The remaining game features and individual risk profiles (including gender, age, weight, BMI, diabetes type, and diabetes duration) were ranked by classifiers according to the estimated importance supported by R Caret library (50). The top-ten ranked variables were utilized as final predictors for the classification model.

Due to the exploratory nature of this study and the limited amount of data, we did not additionally divide the test data set from the entire data set. However, a ten-fold ten repeats cross-validation was utilized during training to avoid overfitting and derive a more accurate estimate of the model performance. This statistical method repeatedly divided the training data set into ten subsets of approximately equal size ten times. Each subset contained the same proportion of labels as the complete data set. Nine of the ten subsets were utilized in the model training, while the remaining subset was used for validation. Multiple classifiers including random forest (RF), lasso and elastic-net regularized generalized linear models (GLMNET), support vector machines (SVM), gradient boosting model (GBM), neural network (NN), and penalized logistic regression (PLR) were tested during modeling. The area under the receiver-operating-characteristic curve (AUC-ROC) was selected as the performance metric to compare the models trained with different feature combinations.

## Results

### Characteristics of study participants

**Figure 2:**
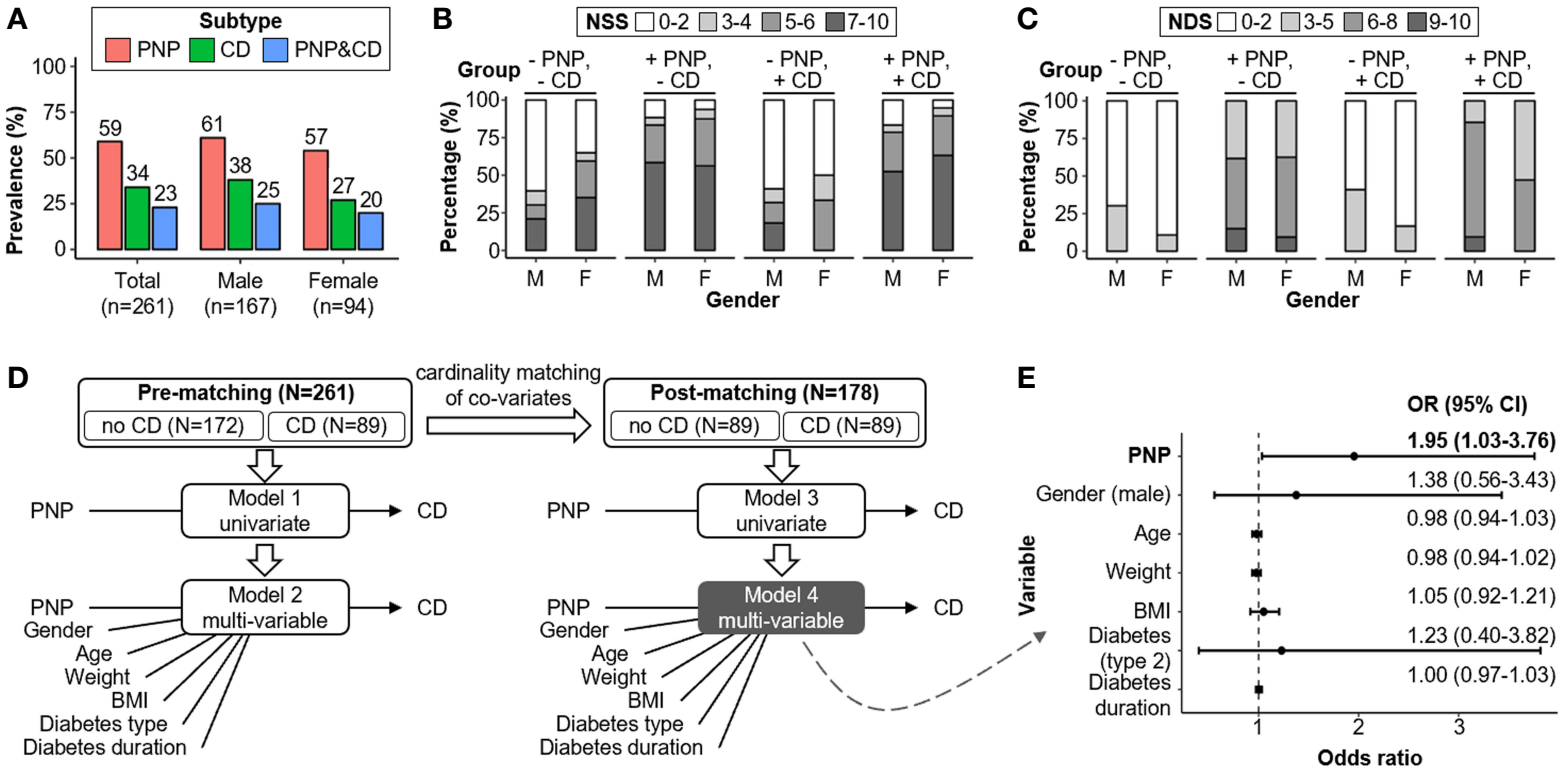
Comparative analysis of PNP and CD co-/prevalence and associated risk factors. **(A)** Cohort-wide PNP and CD prevalence rates segregated by gender. **(B, C)** Male-female disparity for NSS and NDS. **(D)** Regression analyses of risk factors for CD before and after co-variates matching (i.e., gender, age, weight, BMI, diabetes type and diabetes duration). **(E)** CD Risk factors evaluation in the post-matching Subcohort (N=178) with adjustments for gender, age, weight, BMI, type of diabetes, and duration of diabetes (model 4). BMI, body-mass index; CD, cognitive dysfunction; CI, confidence interval; NDS, neuropathy disability score; NSS, neuropathy symptoms score; PNP, peripheral neuropathy.

The study cohort consisted of 261 patients with the diagnosis of diabetes mellitus (age >50 years). These were assigned into four groups according to the absence and/or presence of “clinical” CD and PNP, as defined in the Methods section. Patient characteristics and clinical findings are summarized in **Table 1**. Totally, 80 out of 261 (31%) patients presented without PNP and without CD (-PNP, -CD). 92 (35%) and 28 (11%) patients were labeled as having only PNP (+PNP, -CD) or CD (-PNP, +CD), respectively. The combination of CD and PNP was detected in 61 (23%) patients (+PNP, +CD). The gender distribution skewed toward males in all groups, especially among patients with CD only (~ 4:1). Patients with PNP and/or CD were older and had longer diabetes duration in comparison to patients without PNP and without CD. The overall prevalence of CD and PNP in the cohort is 59% (n=153) and 34% (n=89), respectively (**Figure 2A**). The prevalence and co-prevalence of CD and PNP were higher in male patients compared to female participants. Female patients were more likely to suffer from severe neuropathic symptoms than male participants, however were diagnosed with fewer neuropathic deficits by clinical examinations for PNP (**Figures 2B, C**).

**Table 1:** Demographic and clinical profiles of study participants.

Diabetes groups	-PNP, -CD	+PNP, -CD	-PNP, +CD	+PNP, +CD	*P*
N=261	(N=80)	(N=92)	(N=28)	(N=61)	
Gender					0.08
Female (N=94, 36%)	37 (46.2%)	32 (34.8%)	6 (21.4%)	19 (31.1%)	
Male (N=167, 64%)	43 (53.8%)	60 (65.2%)	22 (78.6%)	42 (68.9%)	
Age (years)	65.0 (11.0)	69.0 (9.0)	68.5 (13.5)	71.0 (9.0)	**0.004**
Weight (kg)	85.5 (20.2)	88.0 (20.0)	84.5 (18.2)	87.0 (24.0)	0.22
BMI (kg/m^2^)	28.2 (7.3)	29.5 (7.1)	26.9 (5.0)	29.6 (7.3)	0.19
Type of diabetes					0.07
Type 1 (N=51, 19.5%)	17 (21.2%)	24 (26.1%)	2 (7.1%)	8 (13.1%)	
Type 2 (N=210, 80.5%)	63 (78.8%)	68 (73.9%)	26 (92.9%)	53 (86.9%)	
Duration of diabetes	10.0 (11.2)	18.0 (21.0)	10.0 (18.2)	16.0 (12.0)	**0.03**
NSS	3.0 (7.0)	7.0 (3.0)	2.0 (5.0)	7.0 (3.0)	**<.001**
NDS	2.0 (2.0)	6.0 (4.0)	2.0 (1.5)	6.0 (2.0)	**<.001**
MoCA	28.0 (2.0)	27.0 (2.0)	23.0 (3.0)	24.0 (3.0)	**<.001**

Categorical variables are presented as n (%). Mean (standard deviation [SD]) and median (interquartile range [IQR]) are used for description of normally and non-normally distributed data, respectively. BMI, body-mass index; CD, cognitive dysfunction; MoCA, Montreal Cognitive Assessment; NDS, neuropathy disability score; NSS, neuropathy symptoms; PNP, peripheral neuropathy.

Bold numbers indicate that level of significance is reached with p values below 0.05.

### Association of PNP with CD

We performed cardinality matching between the groups -CD versus +CD on the entire cohort (n=261) and yielded a matched Subcohort with 89 patients for each group (n=178; **Figure 2D**; **Table 2**). Unbalanced covariates were adjusted through this step including gender, age, weight, BMI, diabetes type and diabetes duration. Logistic regression analyses before and after group matching suggested that the presence of PNP and high NDS were positively associated with the occurrence of CD (PNP: adjusted OR=1.95; 95% CI: 1.03-3.76; *P*=0.04; NDS: adjusted OR=1.16; 95% CI: 1.03-1.32; *P*=0.02; **Figure 2E**; **Table 3**). In addition, “clinical” findings on large fiber neuropathy revealed a significant association with CD, e.g. severe vibration perception dysfunction (left foot: adjusted OR=2.98; 95% CI: 1.22-7.77; *P*=0.02), absent ankle reflex (right foot: adjusted OR=3.07; 95% CI: 1.41-6.90; *P*=0.005), positive correlation with 10-g monofilament test results (left foot: adjusted OR=2.31; 95% CI: 1.06-5.21; *P*=0.04) (**Supplementary Table 1**). Clinical findings indicating small fiber neuropathy such as temperature and pain sensation (pinprick results) did not correlate with the presence of CD. Thus, the small fiber damage mechanisms are likely distinct from the ones taking place within the brain as well as the ones for the large fibers.

**Table 2:** Patient characteristics and clinical profiles before and after covariate matching according to CD distribution.

Characteristic	Pre-Matching				Post-Matching			
	Total	no CD	CD	*P*	Total	no CD	CD	*P*
	(N=261)	(N=172)	(N=89)		(N=178)	(N=89)	(N=89)	
Gender								
Female	94 (36.0%)	69 (40.1%)	25 (28.1%)	0.08	52 (29.2%)	27 (30.3%)	25 (28.1%)	0.87
Male	167 (64.0%)	103 (59.9%)	64 (71.9%)		126 (70.8%)	62 (69.7%)	64 (71.9%)	
Age (years)	68.0 (11.0)	67.0 (10.2)	70.0 (11.0)	**0.02**	69.2 (7.4)	69.3 (7.0)	69.0 (7.7)	0.75
Weight (kg)	86.0 (20.5)	87.5 (18.2)	86.0 (23.0)	0.95	87.5 (25.0)	88.0 (27.0)	86.0 (23.0)	0.58
BMI (kg/m^2^)	28.9 (7.9)	29.0 (7.6)	28.7 (7.6)	0.72	29.4 (8.3)	30.1 (8.6)	28.7 (7.6)	0.66
Diabetes type								
Type 1	51 (19.5%)	41 (23.8%)	10 (11.2%)	**0.02**	20 (11.2%)	10 (11.2%)	10 (11.2%)	1.00
Type 2	210 (80.5%)	131 (76.2%)	79 (88.8%)		158 (88.8%)	79 (88.8%)	79 (88.8%)	
Diabetes duration	13.0 (17.0)	12.0 (18.0)	14.0 (15.0)	0.80	14.0 (17.0)	13.0 (18.0)	14.0 (15.0)	0.65
NSS	6.0 (7.0)	6.0 (8.0)	6.0 (6.0)	0.91	6.0 (6.0)	6.0 (7.0)	6.0 (6.0)	0.71
NDS	4.0 (4.0)	4.0 (4.0)	6.0 (3.0)	**0.002**	4.0 (4.0)	4.0 (4.0)	6.0 (3.0)	**0.01**
PNP presence								
no PNP	108 (41.4%)	80 (46.5%)	28 (31.5%)	0.03	68 (38.2%)	40 (44.9%)	28 (31.5%)	0.09
PNP	153 (58.6%)	92 (53.5%)	61 (68.5%)		110 (61.8%)	49 (55.1%)	61 (68.5%)	

Mean (standard deviation [SD]) and median (interquartile range [IQR]) are used for description of normally and non-normally distributed data, respectively. BMI, body-mass index; CD, cognitive dysfunction; MoCA, Montreal Cognitive Assessment; NDS, neuropathy disability score; NSS, neuropathy symptoms; PNP, peripheral neuropathy.

Bold numbers indicate that level of significance is reached with p values below 0.05.

**Table 3:** Analyses of the association of PNP with CD before and after covariate matching.

Characteristic	Pre-Matching (N=261)				Post-Matching (N=178)			
	Model 1 (univariate)		Model 2 (multi-variable)		Model 3 (univariate)		Model 4 (multi-variable)	
	OR (95% CI)	*P*	OR (95% CI)	*P*	OR (95% CI)	*P*	OR (95% CI)	*P*
PNP presence								
no PNP	ref		ref		ref		ref	
PNP	1.89 (1.11-3.28)	**0.02**	1.87 (1.06-3.36)	**0.03**	1.78 (0.97-3.30)	0.07	1.95 (1.03-3.76)	**0.04**
NSS								
Total	1.01 (0.93-1.09)	0.86	1.00 (0.93-1.09)	0.94	1.01 (0.92-1.10)	0.84	1.01 (0.93-1.11)	0.75
Normal (0-2)	ref		ref		ref		ref	
Mild (3-4)	1.09 (0.34-3.24)	0.88	1.31 (0.39-4.15)	0.65	0.96 (0.26-3.48)	0.95	0.96 (0.26-3.61)	0.96
Moderate (5-6)	1.11 (0.53-2.28)	0.79	1.14 (0.53-2.45)	0.73	0.81 (0.35-1.82)	0.60	0.83 (0.36-1.92)	0.67
Severe (7-10)	1.01 (0.54-1.91)	0.97	0.97 (0.51-1.89)	0.94	1.04 (0.50-2.17)	0.92	1.08 (0.51-2.31)	0.84
NDS								
Total	1.14 (1.04-1.26)	**0.008**	1.13 (1.02-1.26)	**0.02**	1.13 (1.01-1.27)	**0.03**	1.16 (1.03-1.32)	**0.02**
Normal (0-2)	ref		ref		ref		ref	
Mild (3-5)	1.75 (0.87-3.58)	0.12	1.63 (0.79-3.45)	0.19	1.16 (0.53-2.54)	0.72	1.32 (0.59-2.98)	0.50
Moderate (6-8)	3.19 (1.65-6.36)	**<.001**	3.33 (1.61-7.12)	**0.0014**	3.36 (1.52-7.64)	**0.003**	4.04 (1.73-9.88)	**0.002**
Severe (9-10)	1.17 (0.30-3.83)	0.81	0.98 (0.24-3.41)	0.98	0.89 (0.21-3.39)	0.87	1.03 (0.23-4.12)	0.97

Model 1&3: Univariate regression analysis without adjustments for confounding factors. Model 2&4: Multiple variable regression analysis with adjustments for gender, age, weight, BMI, type of diabetes, and duration of diabetes. CD, cognitive dysfunction; NDS, neuropathy disability score; NSS, neuropathy symptoms score; PNP, peripheral neuropathy.

Bold numbers indicate that level of significance is reached with p values below 0.05.

### Subgroup analyses

We initially performed subgroup analyses by gender and found female patients with PNP more likely developed CD than those without PNP (adjusted OR=5.04; 95% CI: 1.22-24.97; *P*=0.03, see **Supplementary Table 2**). However, no significant correlation between CD and PNP occurrence was determined in male participants. Only the NDS of male patients revealed a positive correlation with CD (adj. OR=1.17; 95% CI: 1.02-1.36; *P*=0.03, see **Supplementary Table 3**). These findings indicate that gender-specific mechanisms take place in the development of CD as well as PNP, possibly due to endocrine or genetic differences.

### Game feature analysis

**Figure 3:**
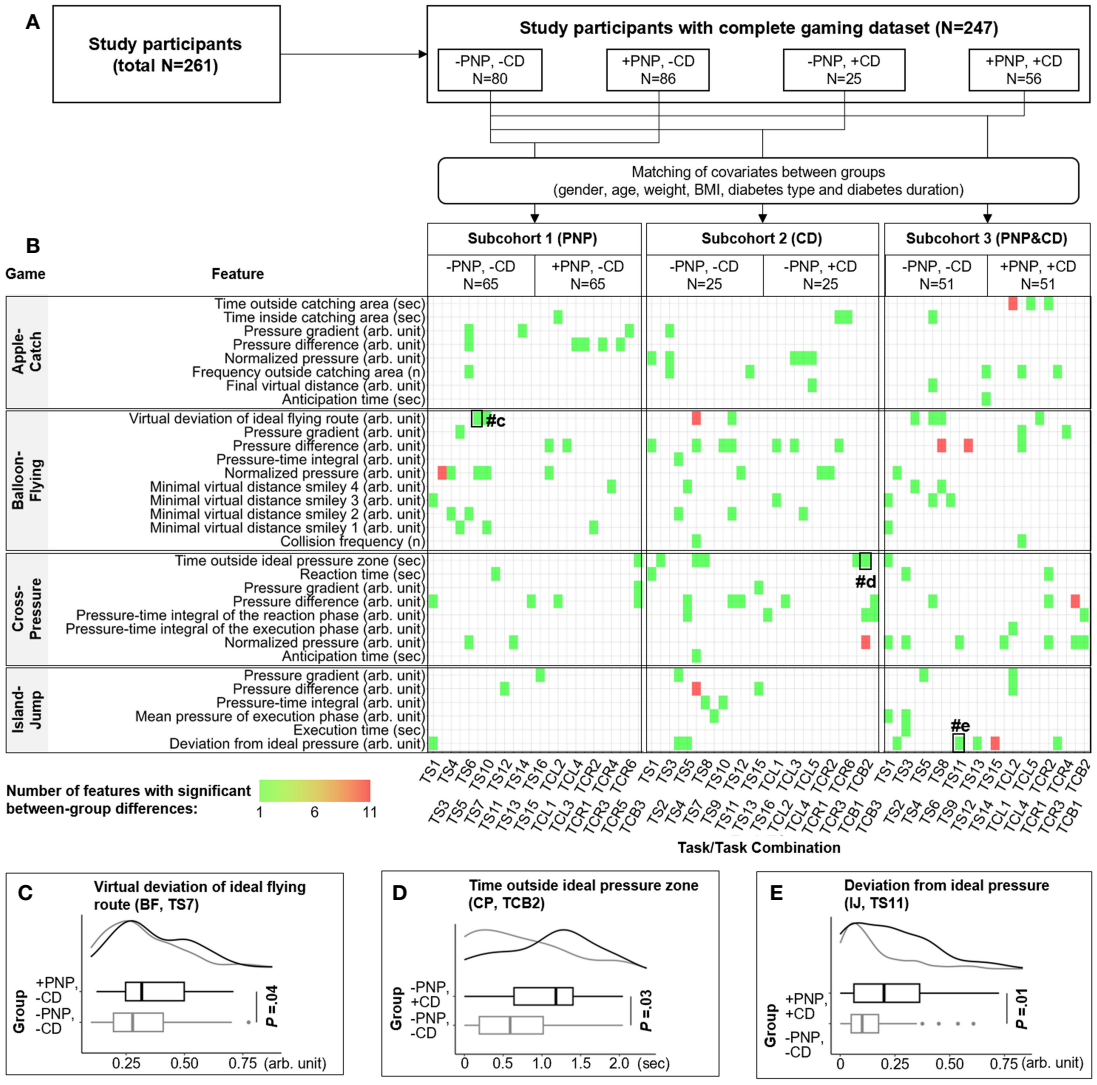
Identification of game features specific for PNP and CD. **(A)** 261 participants were divided into four groups by the occurrence of CD or PNP. The first group (-PNP, -CD) is a reference group including patients without CD and without PNP. Subcohorts 1-3 with matched covariates (age, gender, etc.) were generated to identify game features that were correlated with CD, PNP, or both disorders. All extracted game features were compared between groups within each subcohort. **(B)** The distribution of extracted game features that are significantly correlated with CD (n=59), PNP (n=40), or both (n=59). The colour of the rectangle indicates the number of features from one to eleven. The horizontal and vertical axis denote the name of the game feature and the corresponding source (i.e., a particular game task or combination of tasks), respectively. **(C–E)** examples of game features with significant between-group differences. CD, cognitive decline; PNP, peripheral neuropathy; TCL, task combination for the left foot; TCR, task combination for the right foot; TCB, task combination for both feet; TS, game task.

All patients (n=261) performed the game session immediately after the clinical examination (**Figure 3**). 247 completed gaming data sets for all four games that were included in the analyses. Three Subcohorts were built to explore the relationship between game performance and PNP/CD conditions. The exact proportion of patients is presented in **Figure 3A**. The patient group without PNP and without CD (-PNP, -CD) served as reference group in the gaming data analyses. The covariates gender, age, weight, BMI, diabetes type, and diabetes duration were initially matched between groups.

For each gaming data set (each person), 5,244 game parameters were extracted according to the proposed feature extraction methodologies (**Supplementary Figure 6**). After pre-processing, 1,223 independent game features were compared between groups. Overall, three different lists of predictive game features were extracted that are significantly correlated with +CD (n=59), +PNP (n=40), or both combined (n=59). The distribution of these features is visualized with a heatmap in **Figure 3B**, with three examples presented in **Figures 3C–E**. Thus, individual features are linked with the respective clinical classifiers, which translates into distinct play patterns.

### Classification models for CD and PNP

**Figure 4:**
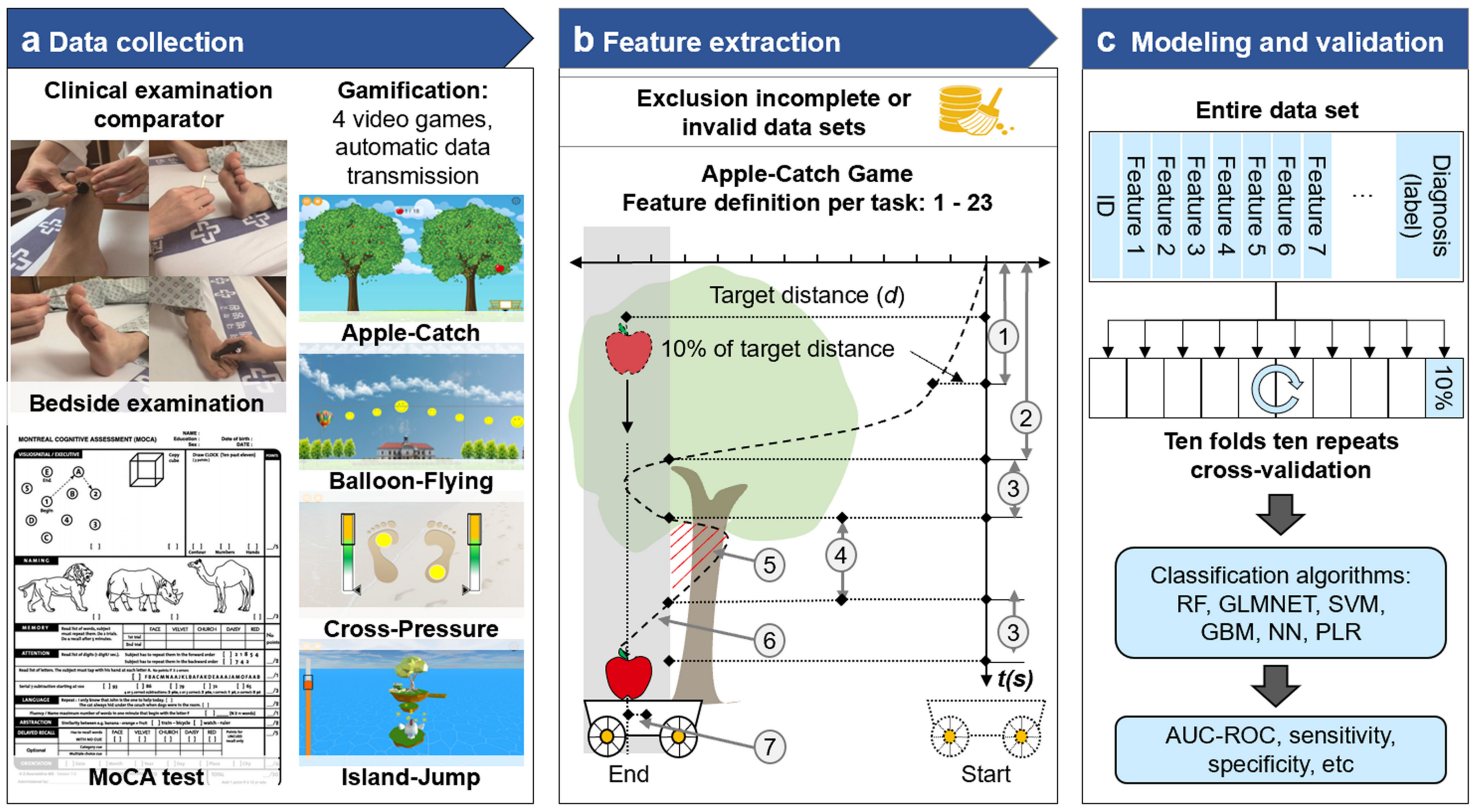
Flow diagram of the development of game-based predictive models for PNP and CD. **(A)** Clinical examination, MOCA testing and performance of 4 video games were transferred to a centralized data bank. **(B)** Incomplete or invalid data sets were excluded and feature extraction was performed with pre-defined game parameters. **(C)** The modeling included the entire data sets and clinical labels. Commonly used classification algorithms yielded findings on AUC-ROC, sensitivity and specificity. AUC-ROC, Area under the receiver operating characteristic curve; GBM, gradient boosting model; GLMNET, lasso and elastic-net regularized generalized linear models; MoCA, Montreal Cognitive Assessment; NN, neural network; RF, random forest; SVM, support vector machines; PLR, penalized logistic regression.

**Figure 5:**
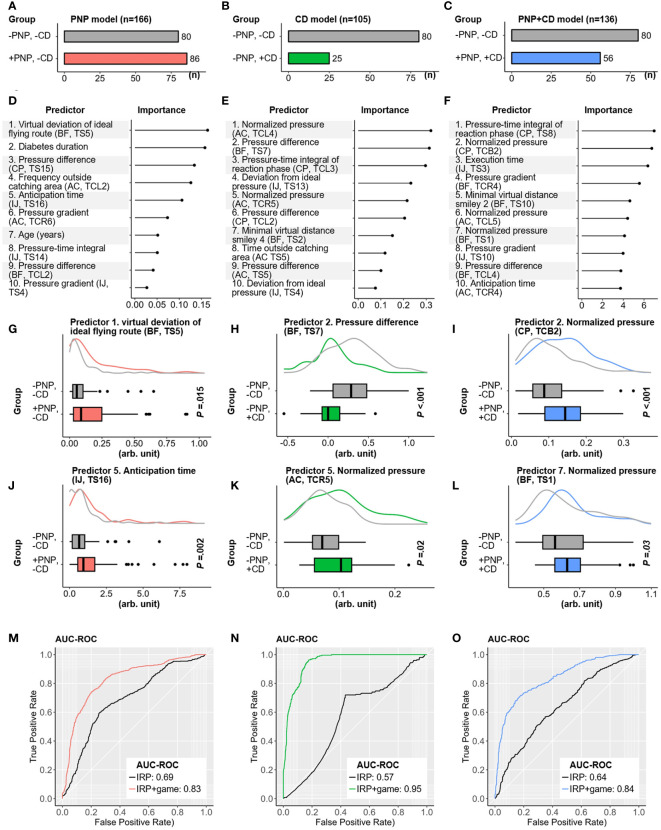
Contribution of selected game features for discrimination by algorithms and performance of classification models for CD, PNP, and CD/PNP combined. **(A–C)** Distribution of CD, PNP, and both disorders in three Subcohorts. **(D–F)** Top ten ranked game features and individual risk profiles (IRP) associated with pure CD, pure PNP, and both conditions. **(G–L)** Examples of high-ranked predictors for the related classification tasks. **(M–O)** AUC-ROC curves of IRP and IRP+game models in identifying pure CD, pure PNP, and both disorders combined. AC, Apple-Catch game; AUC-ROC, Area under the receiver operating characteristic curve; BF, Balloon-Flying game; CD, cognitive dysfunction; CP, Cross-Pressure game; IJ, Island-Jump game; IRP, individual risk profiles; PNP, peripheral neuropathy; TCB, task combination for both feet; TCL, task combination for the left foot; TCR, task combination for the right foot; TS, game task.

With the identified features from 247 patients with diabetes, predictive models for PNP and CD were established (see the methodological diagram in **Figure 4**). The utilized data sets and targets of these models are presented in **Figures 5A–C**. Individual risk profiles (IRP: gender, age, weight, BMI, diabetes type, and diabetes duration) and extracted game features were initially ranked according to their importance to the classification models. For each model, 10 top-ranked variables were selected as “final” predictors (**Figures 5D–F**). Examples of predictors for each model are separately visualized in **Figures 5G–L**. As a reference, we also trained a predictive model using only six individual risk profiles (IRP model using age, gender, weight, BMI, diabetes type and diabetes duration as predictors). Its performance was compared with the model with additional game features (IRP+game model). For the differentiation between patients with PNP and without CD (+PNP, -CD) versus patients without PNP and without CD (-PNP, -CD), the obtained GLMNET achieved an AUC-ROC of 0.83 (sensitivity: 78.8%, specificity: 73.4%, **Figure 5M**). Another GLMNET model allowed to distinguish patients without PNP and with CD (-PNP, +CD) from patients without PNP and without CD (-PNP, -CD) with an AUC-ROC of 0.95, a sensitivity of 72.0%, and a specificity of 93.4% (**Figure 5N**). A GBM model yielded an AUC-ROC of 0.85 by classifying between patients with PNP and with CD (+PNP, +CD) versus patients without PNP and without CD (-PNP, -CD) (**Figure 5O**). The obtained IRP+game models revealed a distinct improvement in performance compared to IRP models (red, green, or blue ROC curves versus black ROC curves in **Figures 5M–O**).

## Discussion

According to the multi-variable logistic regression analyses, a significant positive correlation exists between the two complications, CD and PNP, in individuals with diabetes. This finding is in line with others, such as Hicks et al., that PNP is positively associated with mild cognitive impairment or dementia in patients with diabetes aged above 71 years (OR=1.40; 95% CI: 1.03-1.89) (20). However, there was a noted divergence in the correlation between CD and PNP when comparing male and female participants. This observed discrepancy might stem from the unequal gender distribution within the patient groups, particularly in patients with both disorders (+PNP, +CD). This necessitates further investigations to solidify the findings. Additionally, the CD observed in patients diagnosed with diabetes might arise from mechanisms distinct from those impacting peripheral nerves. Even though these complications bear certain resemblances, a plethora of unique factors could potentially determine the preference towards central or peripheral nerve damage. While there is existing research on the connections between diabetes and cognitive decline, there are still several points of contention that need further examination (28, 29). The pathogenesis of PNP has been associated with various mechanisms, some of which have also been implicated in the development of CD, including microvascular vasculopathy, oxidative stress, dyslipidemia, and inflammation (22, 30, 51–53). Matters are complicated by neuropathy damage patterns incited by different insults, such as diabetes, alcohol, vitamin B12 deficiencies (54). The diagnostic criteria for PNP relied exclusively on established “clinical” scales obtained by clinical examination. The reliability and validation of both the NDS and NSS for individuals with CD (MoCA ≤ 25) is unconfirmed. The accuracy of these scales, contingent upon participant comprehension, raises concerns regarding potential biases in the results. Previous research employing these scales on older demographics has often overlooked the implications of undiagnosed cognitive deficits.

Furthermore, we performed a proof-of-concept study and assessed the potential utility of a game-based application in identifying CD and/or PNP among patients diagnosed with diabetes. These games were selected according to an internal review process to quantify vital capacities linked to cognitive and peripheral nerve functionalities, with the results interpreted through advanced AI techniques. Subsequent to our feature extraction process we discerned various game attributes correlating with either PNP, CD, or both. Utilizing the top ten prioritized game features in conjunction with individual risk factors, our classification model exhibited an AUC-ROC of 0.83, 0.95, and 0.85 for detecting pure PNP, pure CD, and concurrent conditions, respectively. Thus, our proof-of-concept study provided an overall positive result with a high accuracy of classification of the patients into the predefined labels. As the individual risk profiles for the patients were calculated before hands a direct comparison was possible. These were exceeded by the video-based game results with feature extraction.

As a gamified health assessment tool, the proposed application promotes enhanced user engagement, provides real-time feedback, mitigates subjective biases, and ensures accurate data acquisition (55). The structured approach of this game-based assessment ensures high standardization and eliminates the need for intervention by healthcare professionals, facilitating ease of completion even for those unfamiliar with gaming. Post-assessment feedback from participants was overwhelmingly favorable. Besides, most commercial video games have been designed for training or rehabilitation to gain physical benefits (e.g., improvements in balance, postural stability, and motor functions) in patients under various pathological conditions, including stroke, cerebral palsy, or Parkinson’s disease (32, 33, 56). Our proposed approach is a straightforward video game application for the detection of peripheral nerve damage. A distinct innovation in our approach is the integration of a sensor-equipped insole as the game’s control mechanism. This not only heightens the immersive gaming experience but also provides an exhaustive evaluation of limb agility and coordination. Unlike many serious games designed for cognitive assessments, which are often merely digital adaptations of traditional paper-pencil tests demanding only tactile interaction (57), our game requires comprehensive player involvement. Aspects including observation, decision-making, reaction time, anticipation, task execution, endurance, and motor coordination are all captured within the game’s performance metrics. This approach mirrors real-world cognitive processes, thereby minimizing any potential discomfort or sense of being scrutinized among participants. Furthermore, the gamified assessment tool can be seamlessly incorporated into a tele-diagnostic infrastructure, facilitating routine self-administered screening for the designated demographic (58). This approach potentially offers an efficient means of allowing the elderly population to monitor their physical capabilities, diminishing reliance on healthcare specialists and specialized diagnostic equipment.

Although our proof-of-concept study provided a positive result, several limitations warrant consideration. First, the patient sample size in this study remains limited, constraining the development of more sophisticated AI models. The model’s performance derived from a ten-fold ten repeats cross-validation, necessitating confirmation through an independent testing data set in subsequent studies. Amassing more data might pave the way for multi-class classifications, potentially discerning four distinct patient groups simultaneously as opposed to multiple binary classifications. Similarly, a larger sample size of patients with type 1 diabetes would allow to test for their relative risk of developing CD or/and PNP compared to type 2 diabetes. Due to the limited sample size we have opted to analyze both conditions together, which may include a bias and heterogeneity of the cohort. Furthermore, the games’ precision in early-stage PNP detection may be debatable, given the lack of comprehensive phenotyping (e.g., minor *vs*. advanced PNP) within the current clinical examination. The inherent variability in neuropathic manifestations and potential fluctuations in patient effort can potentially impact the results. Within the context of CD, recurrent game interactions might induce a training effect, potentially biasing diagnostic conclusions. Additionally, variations in technological adeptness within the elderly demographic could influence game performance. Another aspect to note is the fixed sequence of gaming tasks, which was maintained to uphold standardization. The implications of introducing randomized task sequences warrant exploration in subsequent research endeavors. Ultimately, as the system remains in its prototype phase and the integrated sensor-equipped insoles have yet to reach commercial availability, the reproducibility of our findings by other researchers is limited at present. It will be crucial to endorse broader application. Future research should aim to deploy the game platform in an outpatient context, broadening its reach and assessing its efficacy as a telemedical instrument.

## Conclusions

The proof-of-concept study tested the classification patients with and without PNP and/or CD by video-based games with feature extraction using machine learning. The comparator was the clinical findings by standard routine procedures performed through a physician. The findings indicate a high discriminatory capability through a playful approach. Future studies with larger cohorts may further its discriminative capabilities.

## Data availability statement

The raw data supporting the conclusions of this article will be made available by the authors, without undue reservation.

## Ethics statement

The studies involving humans were approved by Clinical Ethics Committee at the Medical Faculty of the Otto-von-Guericke University Magdeburg. The studies were conducted in accordance with the local legislation and institutional requirements. The participants provided their written informed consent to participate in this study.

## Author contributions

AM: Conceptualization, Data curation, Formal analysis, Investigation, Methodology, Software, Validation, Visualization, Writing – original draft, Writing – review & editing. EL: Data curation, Investigation, Writing – review & editing. JW: Data curation, Investigation, Writing – review & editing. TS: Data curation, Investigation, Writing – review & editing. NE: Data curation, Investigation, Writing – review & editing. IG: Investigation, Writing – review & editing. AB: Investigation, Writing – review & editing. WG: Data curation, Investigation, Writing – review & editing. SS: Formal analysis, Methodology, Writing – review & editing. PM: Conceptualization, Data curation, Formal analysis, Funding acquisition, Investigation, Methodology, Project administration, Resources, Supervision, Writing – original draft, Writing – review & editing.
