# Supplementary material for: Unveiling peripheral neuropathy and cognitive dysfunction in diabetes: an observational and proof-of-concept study with video games and sensor-equipped insoles
Source: Front Endocrinol (Lausanne). 2024 Mar 1;15:1310152. doi: 10.3389/fendo.2024.1310152 (PMC10941030; doi:10.3389/fendo.2024.1310152)
Supplement: Supplementary file 1 [file DataSheet_1.pdf]

Supplementary Material

This appendix has been provided by the authors to give readers additional information about their work.

Supplement to: Ming A, et al. Unveiling peripheral neuropathy and cognitive dysfunction in diabetes: an observational and proof-of-concept study with video games and sensor-equipped insoles.

(PDF updated Dec. 20, 2023)

Table of contents

1 Supplementary Data 2

1.1 Introduction video 2

2 Supplementary Figures 3

2.1 Supplementary Figure 1: Overview of calibration steps. 3

2.2 Supplementary Figure 2. Apple-Catch (AC) game and related feature extraction. 4

2.3 Supplementary Figure 3. Balloon-Flying (BF) game and related feature extraction. 5

2.4 Supplementary Figure 4. Cross-Pressure (CP) game and related feature extraction. 6

2.5 Supplementary Figure 5. Island-Jump (IJ) game and related feature extraction. 7

2.6 Supplementary Figure 6. Presentation of the feature extraction methodology using the Apple-Catch (AC) game as an example. 8

3 Supplementary Tables 9

3.1 Supplementary Table 1. Regression analyses of the association between small-/large fiber nerve deficits and CD before and after covariate matching. 9

3.2 Supplementary Table 2. Subgroup analyses of the association of PNP with CD in female patients. 10

3.3 Supplementary Table 3. Subgroup analyses of the association of PNP and CD in male patients. 11

3.4 Supplementary Table 4. Post-hoc power analyses. 12

4 R packages 13

1 Supplementary Data

1.1 Introduction video

The video is attached with name "Introduction Video EN.mp4".

Alternatively, it is accessible through the following link:

https://osf.io/mksj5/?view_only=30f93f67cfbe4296a7e58e96d0a59df6

2 Supplementary Figures

2.1 Supplementary Figure 1: Overview of calibration steps.

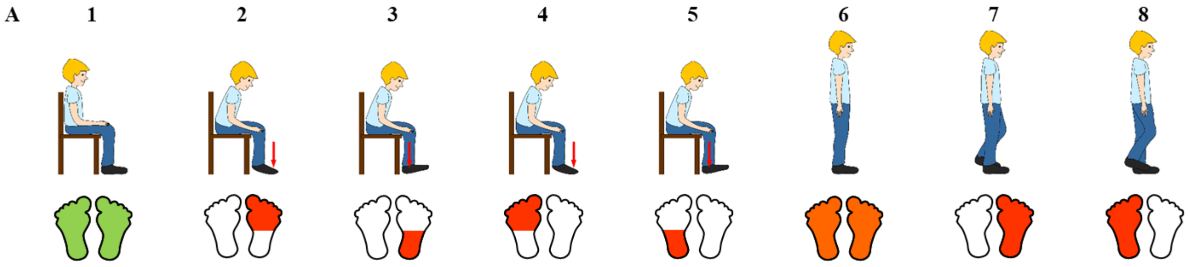

B Calibration step

1 Balanced sitting (5 sec)
2 Maximum pressure on the right forefoot (5 sec)
3 Maximum pressure on the right heel (5 sec)
4 Maximum pressure on the left forefoot (5 sec)
5 Maximum pressure on the left heel (5 sec)
6 Balance standing (5 sec)
7 Standing on the right foot (5 sec)
8 Standing on the left foot (5 sec)

C Normalization algorithm

$$p_n = \frac{p_a - p_{min}}{p_{max} - p_{min}}$$

p_n: *normalized pressure*

p_a: *absolute pressure*

p_{min}: *minimal pressure threshold*

p_{max}: *maximal pressure threshold*

(A) Eight calibration steps are performed before the commencement of the games. These allow to normalize pressure values according to weight and maximum pressure applied by the participants. Minimum and maximum pressure are recorded for all positions, forefeet, and heels. (B) Furthermore, the participants are instructed to stand up for five seconds, keep balance on each foot alone for 5 seconds. (C) An algorithm is applied to normalize these values that are utilized for the steering unit of the insoles. Minimal-maximal normalization transforms all values to the range of 0 to 1.

2.2 Supplementary Figure 2. Apple-Catch (AC) game and related feature extraction.

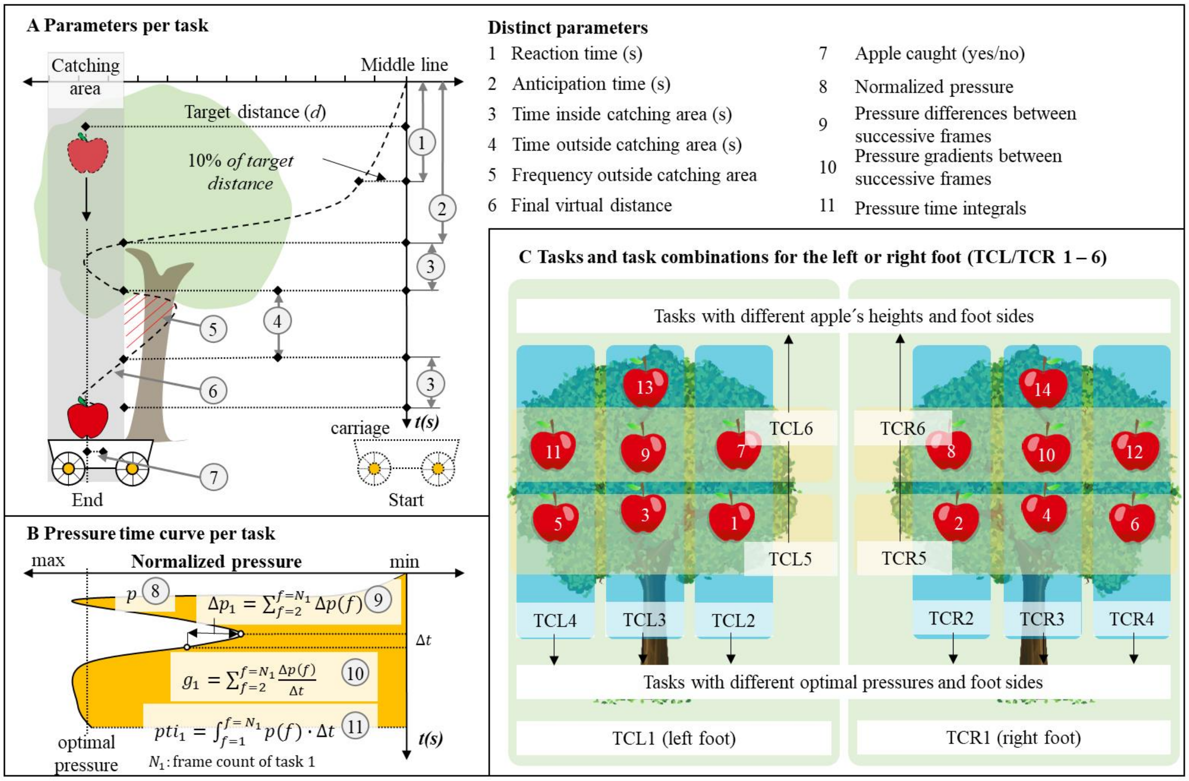

(A) In order to evaluate the performance in apple collection, eleven distinct parameters are defined per task, constituting the basis for data analyses. (B) Pressure-time curve of each task and calculations of pressure differences, pressure gradients, and pressure-time integration. (C) The Apple-Catch game includes fourteen tasks (apples). For feature extraction tasks and task combinations are furthermore defined (TCL/TCR 1–6) for each foot to assess the performance (left/right foot, tasks with different ideal pressures, tasks with different apple heights and foot sides).

2.3 Supplementary Figure 3. Balloon-Flying (BF) game and related feature extraction.

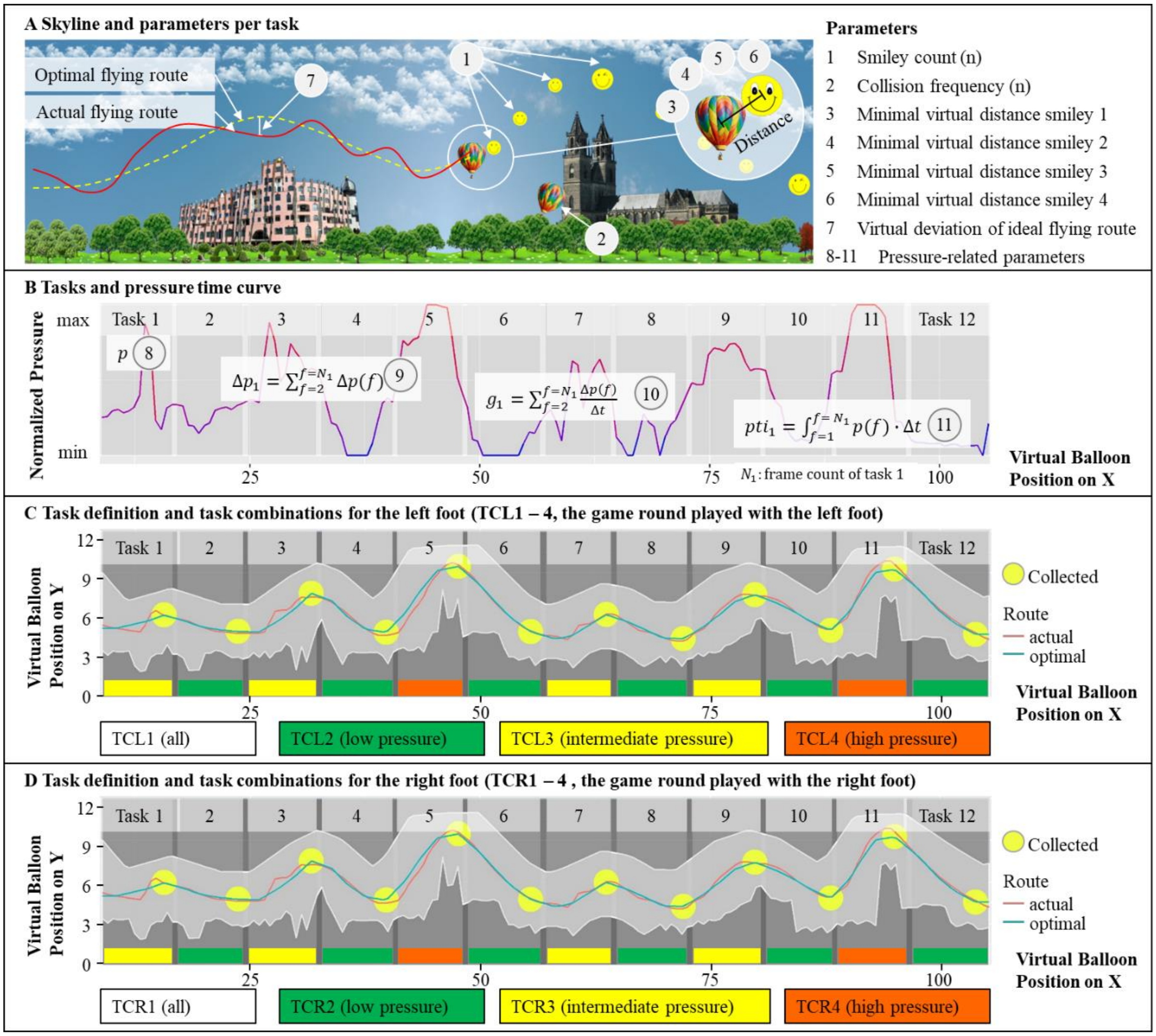

(A) Eleven distinct parameters are extracted following comparison with the predefined optimal flying course of the balloon. These are enlisted and included the overall number of collected smileys (yellow circles), collision frequency, minimum virtual distances balloon to smiley/perfect flight position, and the pressure gradient between consecutive obstacles. (B) The Balloon-Flying parcours consists of twelve distinct tasks. (C&D) Definition of tasks and task combinations for the left and right foot corresponding to low, intermediate, and high-pressure applications (TCL/TCR 1–4).

2.4 Supplementary Figure 4. Cross-Pressure (CP) game and related feature extraction.

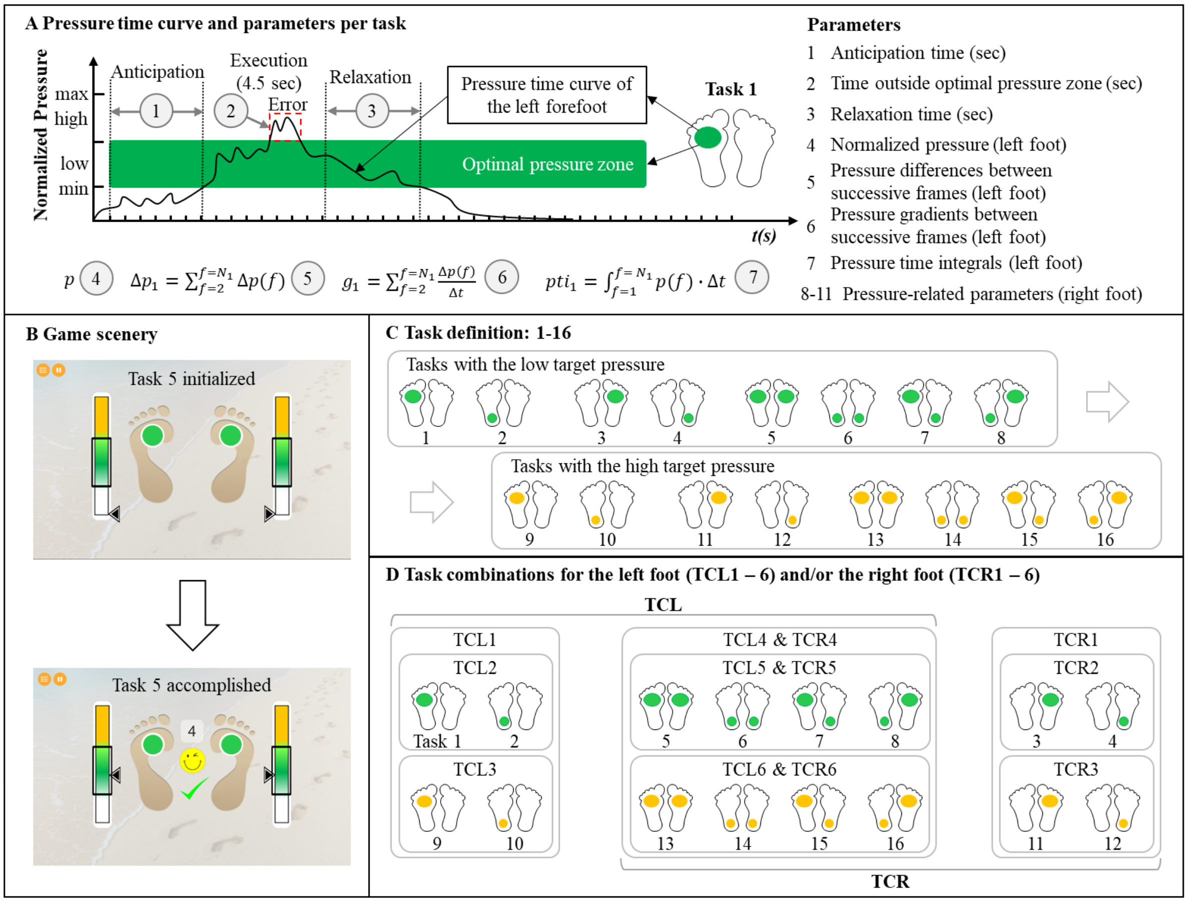

(A) Seven parameters are defined to present the game outcomes per task. These consider the time durations of key game events and pressure gradients. (B) Screenshots of initialized and accomplished game task. (C) The CP game includes sixteen tasks corresponding to 16 combinations of foot areas and ideal pressure levels. (D) Six task combinations for each foot (TCL/TCR 1–6) are defined for extracting features that address player´s performance by different pressure levels (low versus high) and foot areas (left or right foot, forefoot or heel).

2.5 Supplementary Figure 5. Island-Jump (IJ) game and related feature extraction.

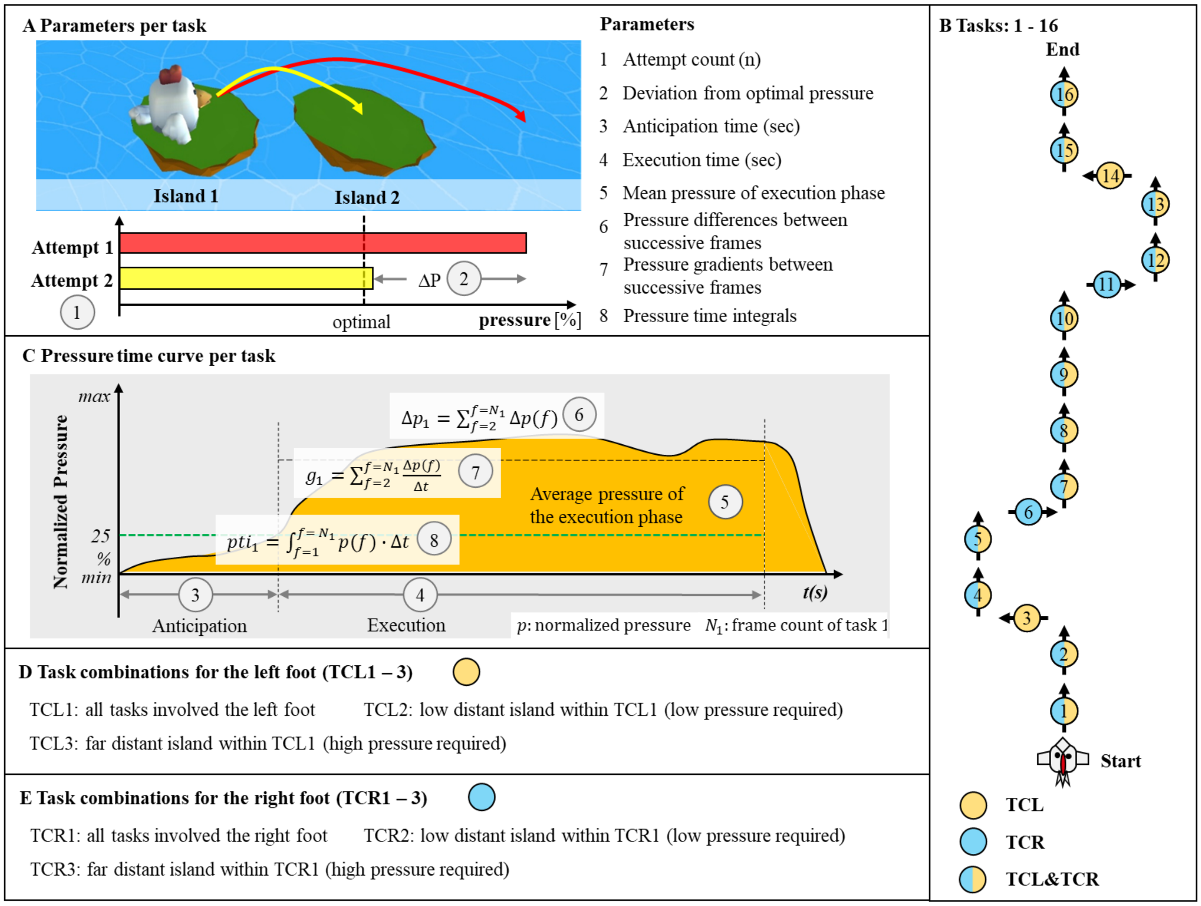

(A) Eight parameters are defined to indicate the game outcomes per task. (B) The IJ game contains sixteen tasks corresponding to 16 islands that the bird has to jump through until reaching home. (C) Pressure-related parameters including normalized pressure, pressure difference, pressure gradient and pressure-time integration of the left/right foot. (D&E) Distribution of task combinations for the left and right foot corresponding to low, intermediate, and high-pressure applications on left, right or both feet.

2.6 Supplementary Figure 6. Presentation of the feature extraction methodology using the Apple-Catch (AC) game as an example.

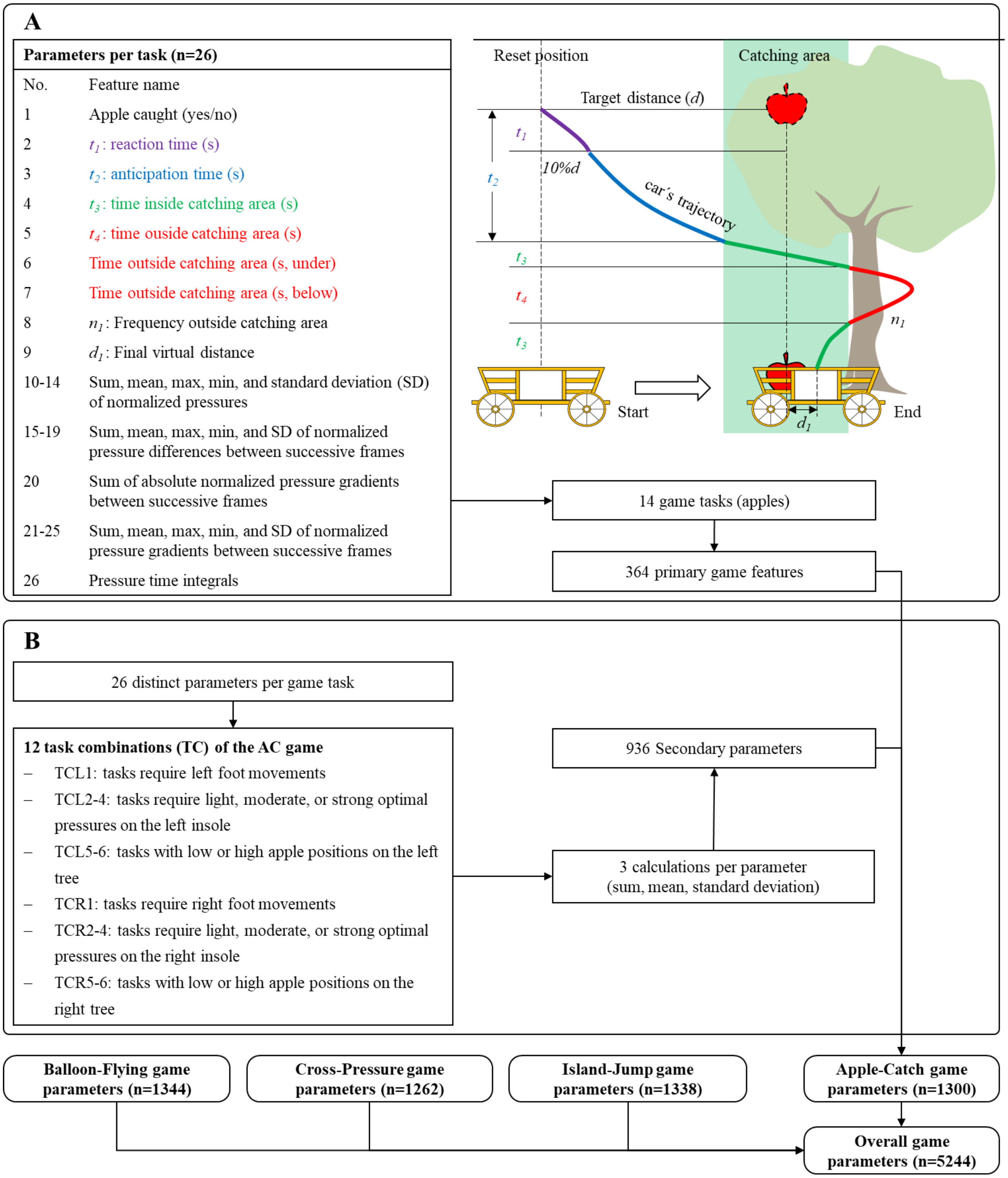

(A) Primary feature extraction for each task in the Apple-Catch game. (B) Secondary feature extraction for task combinations in the Apple-Catch game. TC: task combination; TCL: task combination for the left foot; TCR: task combination for the right foot.

3 Supplementary Tables

3.1 Supplementary Table 1. Regression analyses of the association between small-/large fiber nerve deficits and CD before and after covariate matching.

Model 1&3: Univariate regression analysis without adjustments for confounding factors. Model 2&4: Multiple variable regression analysis with adjustments for gender, age, weight, BMI, type of diabetes, and duration of diabetes. CD: cognitive dysfunction.

Characteristic	Pre-Matching (N=261)				Post-Matching (N=178)			
	Model 1 (univariate)		Model 2 (multi-variable)		Model 3 (univariate)		Model 4 (multi-variable)	
	OR (95%CI)	*P*	OR (95%CI)	*P*	OR (95%CI)	*P*	OR (95%CI)	*P*
Small fiber nerve deficits								
Temperature sensation (L)								
Present	ref		ref		ref		ref	
Reduced/Absent	0.90 (0.53-1.52)	0.70	0.90 (0.52-1.54)	0.69	0.83 (0.45-1.51)	0.54	0.83 (0.45-1.52)	0.54
Temperature sensation (R)								
Present	ref		ref		ref		ref	
Reduced/Absent	0.96 (0.57-1.62)	0.89	0.95 (0.55-1.62)	0.84	1.05 (0.58-1.91)	0.88	1.08 (0.58-2.00)	0.80
Pain sensation (pinprick, L)								
Present	ref		ref		ref		ref	
Reduced/Absent	1.40 (0.74-2.61)	0.29	1.29 (0.67-2.47)	0.44	1.31 (0.64-2.71)	0.47	1.35 (0.64-2.89)	0.42
Pain sensation (pinprick, R)								
Present	ref		ref		ref		ref	
Reduced/Absent	1.38 (0.74-2.54)	0.30	1.24 (0.64-2.38)	0.51	1.30 (0.64-2.65)	0.47	1.42 (0.67-3.06)	0.36
Large fiber nerve deficits								
Vibration perception (L)								
Normal (6-8)	ref		ref		ref		ref	
Mild (5)	3.12 (0.81-11.54)	0.09	2.57 (0.64-9.91)	0.17	2.50 (0.55-12.19)	0.24	2.71 (0.57-13.79)	0.21
Moderate (3-4)	2.89 (1.06-8.07)	**0.04**	2.93 (1.02-8.59)	**0.046**	1.83 (0.60-5.71)	0.29	2.05 (0.65-6.64)	0.22
Severe (0-2)	3.32 (1.62-7.37)	**0.002**	3.21 (1.46-7.58)	**0.005**	2.38 (1.04-5.71)	**0.04**	2.98 (1.22-7.77)	**0.02**
Vibration perception (R)								
Normal (6-8)	ref		ref		ref		ref	
Mild (5)	2.04 (0.54-7.13)	0.27	2.05 (0.51-7.70)	0.29	1.77 (0.39-8.52)	0.46	1.99 (0.42-10.02)	0.39
Moderate (3-4)	1.69 (0.66-4.36)	0.27	1.63 (0.59-4.47)	0.34	1.06 (0.37-3.06)	0.91	1.22 (0.41-3.72)	0.72
Severe (0-2)	2.37 (1.19-5.02)	**0.02**	2.25 (1.04-5.15)	**0.046**	1.63 (0.72-3.81)	0.24	1.94 (0.79-4.91)	0.15
Ankle reflex (L)								
Normal	ref		ref		ref		ref	
Reduced	2.44 (1.30-4.62)	**0.006**	2.39 (1.25-4.64)	**0.009**	2.38 (1.17-4.90)	**0.02**	2.49 (1.21-5.20)	**0.01**
Absent	2.57 (1.35-4.94)	**0.004**	2.55 (1.28-5.17)	**0.008**	2.94 (1.40-6.32)	**0.005**	3.16 (1.45-7.09)	**0.004**
Ankle reflex (R)								
Normal	ref		ref		ref		ref	
Reduced	2.67 (1.42-5.08)	**0.002**	2.63 (1.35-5.18)	**0.005**	2.47 (1.22-5.13)	**0.01**	2.68 (1.29-5.72)	**0.009**
Absent	2.57 (1.36-4.92)	**0.004**	2.54 (1.27-5.16)	**0.009**	2.79 (1.34-5.96)	**0.007**	3.07 (1.41-6.90)	**0.005**
10-g monofilament test (L)								
Present	ref		ref		ref		ref	
Reduced	2.59 (1.33-5.05)	**0.005**	2.68 (1.34-5.42)	**0.006**	2.06 (0.97-4.51)	0.06	2.31 (1.06-5.21)	**0.04**
Absent	1.69 (0.48-5.49)	0.39	1.65 (0.45-5.68)	0.43	1.16 (0.31-4.37)	0.82	1.23 (0.31-4.81)	0.76
10-g monofilament test (R)								
Present	ref		ref		ref		ref	
Reduced	2.20 (1.15-4.21)	**0.02**	2.06 (1.04-4.08)	**0.04**	2.26 (1.06-5.05)	**0.04**	2.50 (1.13-5.80)	**0.03**
Absent	1.43 (0.42-4.48)	0.54	1.33 (0.37-4.39)	0.64	1.18 (0.31-4.43)	0.80	1.27 (0.33-4.95)	0.73

3.2 Supplementary Table 2. Subgroup analyses of the association of PNP with CD in female patients.

Model 1&3: Univariate regression analysis without adjustments for confounding factors. Model 2&4: Multiple variable regression analysis with adjustments for gender, age, weight, BMI, type of diabetes, and duration of diabetes. CD: cognitive dysfunction; NDS: neuropathy disability score; PNP: peripheral neuropathy.

Characteristic	Pre-Matching (N=94)				Post-Matching (N=50)			
	Model 1 (univariate)		Model 2 (multi-variable)		Model 3 (univariate)		Model 4 (multi-variable)	
	OR (95%CI)	*P*	OR (95%CI)	*P*	OR (95%CI)	*P*	OR (95%CI)	*P*
PNP presence								
no PNP	ref		ref		ref		ref	
PNP	3.66 (1.36-11.08)	**0.01**	4.18 (1.32-14.94)	**0.02**	2.92 (0.90-10.33)	0.08	5.04 (1.22-24.97)	**0.03**
NDS	1.17 (0.98-1.41)	0.09	1.20 (0.96-1.52)	0.12	1.04 (0.84-1.29)	0.75	1.09 (0.84-1.44)	0.51

3.3 Supplementary Table 3. Subgroup analyses of the association of PNP and CD in male patients.

Model 1&3: Univariate regression analysis without adjustments for confounding factors. Model 2&4: Multiple variable regression analysis with adjustments for gender, age, weight, BMI, type of diabetes, and duration of diabetes. CD: cognitive dysfunction; NDS: neuropathy disability score; PNP: peripheral neuropathy.

Characteristic	Pre-Matching (N=167)				Post-Matching (N=128)			
	Model 1 (univariate)		Model 2 (multi-variable)		Model 3 (univariate)		Model 4 (multi-variable)	
	OR (95%CI)	*P*	OR (95%CI)	*P*	OR (95%CI)	*P*	OR (95%CI)	*P*
PNP presence								
no PNP	ref		ref		ref		ref	
PNP	1.37 (0.72-2.64)	0.34	1.54 (0.77-3.14)	0.23	1.58 (0.78-3.25)	0.21	1.71 (0.81-3.67)	0.16
NDS	1.11 (0.99-1.25)	0.08	1.13 (1.00-1.28)	0.06	1.15 (1.00-1.32)	**0.047**	1.17 (1.02-1.36)	**0.03**

3.4 Supplementary Table 4. Post-hoc power analyses.

Sample Size	Pre-Matching N=261	Post-Matching N=178	
Ratio of Groups			
with PNP	N=108 (41%)	N=68 (38%)	
without PNP	N=153 (59%)	N=110 (62%)	
Odds ratio (OR)	PNP is positively associated with CD in diabetes (OR=1.40; 95% CI: 1.03-1.89)		Hicks CW, et al. Associations of Peripheral Neuropathy Defined by Monofilament Insensitivity with Mild Cognitive Impairment and Dementia in Older Adults. Dement Geriatr Cogn Disord. 2022
Effect size	0.19		Approximated Cohen's d for OR=1.40.
Adjusted effect size	0.38	0.38	Adjust the effect size for the group ratio
Alpha Level (Type I error rate)	0.05	0.05	An approximate conversion from the log odds ratio to Cohen's d is given by $Cohen's\ d = Log\ (OR) \times \sqrt{3}/\pi$ This approximation is based on the assumption that the underlying variables are normally distributed.
Power	99%	95%	Desired power level, usually 80% or 90%. Power is the probability of correctly rejecting the null hypothesis when it is false.
Statistical Software	R (v4.2.1) and "pwr" package (v1.3-0)		

CD: cognitive dysfunction; PNP: peripheral neuropathy.

4 R packages

abind (Version: 1.4-5), alphavantager (Version: 0.1.3), anytime (Version: 0.3.9), arrangements (Version: 1.1.9), askpass (Version: 1.1), assertthat (Version: 0.2.1), backports (Version: 1.4.1), base64enc (Version: 0.1-3), beeswarm (Version: 0.4.0), BH (Version: 1.81.0-1), bit (Version: 4.0.5), bit64 (Version: 4.0.5), bitops (Version: 1.0-7), blob (Version: 1.2.4), bmp (Version: 0.3), brew (Version: 1.0-8), brio (Version: 1.1.3), broom (Version: 1.0.4), bslib (Version: 0.4.2), cachem (Version: 1.0.8), callr (Version: 3.7.3), car (Version: 3.1-2), carData (Version: 3.0-5), caret (Version: 6.0-94), caTools (Version: 1.18.2), CBPS (Version: 0.23), cellranger (Version: 1.1.0), checkmate (Version: 2.2.0), chk (Version: 0.8.1), CircStats (Version: 0.2-6), cli (Version: 3.6.0), clipr (Version: 0.8.0), clock (Version: 0.6.1), cobalt (Version: 4.5.1), coin (Version: 1.4-2), colorspace (Version: 2.1-0), combinat (Version: 0.0-8), common (Version: 1.0.5), commonmark (Version: 1.9.0), CompQuadForm (Version: 1.4.3), conflicted (Version: 1.2.0), corrplot (Version: 0.92), cowplot (Version: 1.1.1), cpp11 (Version: 0.4.3), crayon (Version: 1.5.2), credentials (Version: 1.3.2), crosstalk (Version: 1.2.0), curl (Version: 5.0.0), cvAUC (Version: 1.1.4), data.table (Version: 1.14.8), DBI (Version: 1.1.3), dbplyr (Version: 2.3.2), deldir (Version: 1.0-6), dendextend (Version: 1.17.1), DEoptimR (Version: 1.0-13), Deriv (Version: 4.1.3), desc (Version: 1.4.2), devtools (Version: 2.4.5), dfidx (Version: 0.0-5), diagram (Version: 1.6.5), dials (Version: 1.2.0), DiceDesign (Version: 1.9), diffobj (Version: 0.3.5), digest (Version: 0.6.31), distances (Version: 0.1.9), distributional (Version: 0.3.2), doParallel (Version: 1.0.17), dotCall64 (Version: 1.0-2), downlit (Version: 0.4.2), downloader (Version: 0.4), dplyr (Version: 1.1.2), DT (Version: 0.27), dtplyr (Version: 1.3.1), dtw (Version: 1.23-1), e1071 (Version: 1.7-13), egg (Version: 0.4.5), elasticnet (Version: 1.3), ellipse (Version: 0.4.5), ellipsis (Version: 0.3.2), emmeans (Version: 1.8.5), estimability (Version: 1.4.1), evaluate (Version: 0.21), exactRankTests (Version: 0.8-35), factoextra (Version: 1.0.7), FactoMineR (Version: 2.8), fansi (Version: 1.0.4), farver (Version: 2.1.1), fastmap (Version: 1.1.1), fields (Version: 14.1), flashClust (Version: 1.01-2), flsa (Version: 1.5.2), fmsb (Version: 0.7.5), fmtr (Version: 1.5.9), fontawesome (Version: 0.5.1), forcats (Version: 1.0.0), foreach (Version: 1.5.2), forecast (Version: 8.21), Formula (Version: 1.2-5), fracdiff (Version: 1.5-2), fs (Version: 1.6.2), furrr (Version: 0.3.1), future (Version: 1.32.0), future.apply (Version: 1.10.0), gam (Version: 1.22-2), gargle (Version: 1.4.0), gbm (Version: 2.1.8.1), gdata (Version: 2.19.0), generics (Version: 0.1.3), gert (Version: 1.9.2), GGally (Version: 2.1.2), ggbeeswarm (Version: 0.7.2), ggdist (Version: 3.2.1), gghalves (Version: 0.1.4), ggnewscale (Version: 0.4.8), ggplot2 (Version: 3.4.2), ggpubr (Version: 0.6.0), ggradar (Version: 0.2), ggrepel (Version: 0.9.3), ggsci (Version: 3.0.0), ggsignif (Version: 0.6.4), ggtext (Version: 0.1.2), ggthemes (Version: 4.2.4), gh (Version: 1.4.0), gitcreds (Version: 0.1.2), glmnet (Version: 4.1-7), globals (Version: 0.16.2), glue (Version: 1.6.2), gmodels (Version: 2.18.1.1), gmp (Version: 0.7-1), googleAuthR (Version: 2.0.1), googledrive (Version: 2.1.0), googlesheets4 (Version: 1.1.0), gower (Version: 1.0.1), GPfit (Version: 1.0-8), gplots (Version: 3.1.3), greybox (Version: 1.0.8), gridExtra (Version: 2.3), gridSVG (Version: 1.7-5), gridtext (Version: 0.1.5), gtable (Version: 0.3.3), gtools (Version: 3.9.4), hardhat (Version: 1.3.0), haven (Version: 2.5.2), HDInterval (Version: 0.2.4), highr (Version: 0.10), Hmisc (Version: 5.0-1), hms (Version: 1.1.3), htmlTable (Version: 2.4.1), htmltools (Version: 0.5.5), htmlwidgets (Version: 1.6.2), httpuv (Version: 1.6.9), httr (Version: 1.4.6), httr2 (Version: 0.2.2), ids (Version: 1.0.1), ieugwasr (Version: 0.1.5), igraph (Version: 1.4.2), imager (Version: 0.42.19), infer (Version: 1.0.4), ini (Version: 0.3.1), insight (Version: 0.19.1), interp (Version: 1.1-4), inum (Version: 1.0-5), ipred (Version: 0.9-14), isoband (Version: 0.2.7), iterators (Version: 1.0.14), iterpc (Version: 0.4.2), jpeg (Version: 0.1-10), jquerylib (Version: 0.1.4), jsonlite (Version: 1.8.4), jtools (Version: 2.2.1), kernlab (Version: 0.9-32), km.ci (Version: 0.5-6), KMsurv (Version: 0.1-5), knitr (Version: 1.42), labeling (Version: 0.4.2), laeken (Version: 0.5.2), lars (Version: 1.3), later (Version: 1.3.1), latticeExtra (Version: 0.6-30), lava (Version: 1.7.2.1), lavaan (Version: 0.6-

15), lazyeval (Version: 0.2.2), lcmm (Version: 2.1.0), leaps (Version: 3.1), lhs (Version: 1.1.6), libcoin (Version: 1.0-9), LiblineaR (Version: 2.10-22), lifecycle (Version: 1.0.3), listenv (Version: 0.9.0), lme4 (Version: 1.1-33), lmtest (Version: 0.9-40), lubridate (Version: 1.9.2), magrittr (Version: 2.0.3), maps (Version: 3.4.1), marginaleffects (Version: 0.11.1), markdown (Version: 1.6), marqLevAlg (Version: 2.0.8), Matching (Version: 4.10-8), MatchIt (Version: 4.5.3), mathjaxr (Version: 1.6-0), MatrixModels (Version: 0.5-1), matrixStats (Version: 0.63.0), maxstat (Version: 0.7-25), mboost (Version: 2.9-7), memoise (Version: 2.0.1), MendelianRandomization (Version: 0.7.0), meta (Version: 6.2-1), metadat (Version: 1.2-0), metafor (Version: 4.2-0), mime (Version: 0.12), miniUI (Version: 0.1.1.1), minqa (Version: 1.2.5), mitools (Version: 2.4), mlbench (Version: 2.1-3.1), MLeval (Version: 0.3), mlogit (Version: 1.1-1), mnormt (Version: 2.1.1), modeldata (Version: 1.1.0), modelenv (Version: 0.1.1), ModelMetrics (Version: 1.2.2.2), modelr (Version: 0.1.11), modeltools (Version: 0.2-23), mr.raps (Version: 0.2), MRInstruments (Version: 0.3.2), MRMix (Version: 0.1.0), MRPRESSO (Version: 1.0), multcomp (Version: 1.4-23), multcompView (Version: 0.1-9), munsell (Version: 0.5.0), mvtnorm (Version: 1.1-3), nloptr (Version: 2.0.3), nnls (Version: 1.4), nortest (Version: 1.0-4), numDeriv (Version: 2016.8-1.1), OddsPlotty (Version: 1.0.2), openssl (Version: 2.0.6), optmatch (Version: 0.10.6), optweight (Version: 0.2.5), ordinalForest (Version: 2.4-3), osqp (Version: 0.6.0.8), padr (Version: 0.6.2), pander (Version: 0.6.5), parallelly (Version: 1.35.0), parsnip (Version: 1.1.0), party (Version: 1.3-13), partykit (Version: 1.2-20), patchwork (Version: 1.1.2), pbapply (Version: 1.7-0), pbivnorm (Version: 0.6.0), pbkrtest (Version: 0.5.2), penalizedLDA (Version: 1.1), PerformanceAnalytics (Version: 2.0.4), pillar (Version: 1.9.0), pkgbuild (Version: 1.4.0), pkgconfig (Version: 2.0.3), pkgdown (Version: 2.0.7), pkgload (Version: 1.3.2), plotly (Version: 4.10.1), plotROC (Version: 2.3.0), plyr (Version: 1.8.8), png (Version: 0.1-8), polynom (Version: 1.4-1), pracma (Version: 2.4.2), praise (Version: 1.0.0), prettyunits (Version: 1.1.1), pROC (Version: 1.18.0), processx (Version: 3.8.1), prodlim (Version: 2023.03.31), profvis (Version: 0.3.8), progress (Version: 1.2.2), progressr (Version: 0.13.0), promises (Version: 1.2.0.1), proxy (Version: 0.4-27), ps (Version: 1.7.5), pscl (Version: 1.5.5.1), psych (Version: 2.3.3), purrr (Version: 1.0.1), quadprog (Version: 1.5-8), Quandl (Version: 2.11.0), quantmod (Version: 0.4.22), quantreg (Version: 5.95), quantregForest (Version: 1.3-7), quickmatch (Version: 0.2.1), R6 (Version: 2.5.1), RadialMR (Version: 1.0), ragg (Version: 1.2.5), randomForest (Version: 4.7-1.1), randtoolbox (Version: 2.0.4), ranger (Version: 0.15.1), rappdirs (Version: 0.3.3), rbibutils (Version: 2.2.13), rcmdcheck (Version: 1.4.0), RColorBrewer (Version: 1.1-3), Rcpp (Version: 1.0.10), RcppArmadillo (Version: 0.12.2.0.0), RcppEigen (Version: 0.3.3.9.3), RcppProgress (Version: 0.4.2), RcppRoll (Version: 0.3.0), Rdpack (Version: 2.4), readbitmap (Version: 0.1.5), readr (Version: 2.1.4), readxl (Version: 1.4.2), recipes (Version: 1.0.6), rematch (Version: 1.0.1), rematch2 (Version: 2.1.2), remotes (Version: 2.4.2), reprex (Version: 2.0.2), reshape (Version: 0.8.9), reshape2 (Version: 1.4.4), rgenoud (Version: 5.9-0.3), Rglpk (Version: 0.6-5), riingo (Version: 0.3.1), rJava (Version: 1.0-6), rjson (Version: 0.2.21), rlang (Version: 1.1.1), rlemon (Version: 0.2.1), rmarkdown (Version: 2.21), rngWELL (Version: 0.10-9), robustbase (Version: 0.95-1), ROCR (Version: 1.0-11), roxygen2 (Version: 7.2.3), rprojroot (Version: 2.0.3), rsample (Version: 1.1.1), rsq (Version: 2.5), rstatix (Version: 0.7.2), rstudioapi (Version: 0.14), Rtsne (Version: 0.16), rversions (Version: 2.1.2), rvest (Version: 1.0.3), sandwich (Version: 3.0-2), sass (Version: 0.4.6), scales (Version: 1.2.1), scatterplot3d (Version: 0.3-44), scclust (Version: 0.2.3), selectr (Version: 0.4-2), sessioninfo (Version: 1.2.2), sets (Version: 1.0-24), shape (Version: 1.4.6), shiny (Version: 1.7.4), slam (Version: 0.1-50), slider (Version: 0.3.0), smooth (Version: 3.2.1), sourcetools (Version: 0.1.7-1), sp (Version: 1.6-0), spam (Version: 2.9-1), SparseM (Version: 1.81), SQUAREM (Version: 2021.1), stabs (Version: 0.6-4), statmod (Version: 1.5.0), stepPlr (Version: 0.93), stringi (Version: 1.7.12), stringr (Version: 1.5.0), strucchange (Version: 1.5-3), SuperLearner (Version: 2.0-28), survey (Version: 4.2-1), survminer (Version: 0.4.9), survMisc (Version: 0.5.6), sys

(Version: 3.4.1), systemfonts (Version: 1.0.4), testthat (Version: 3.1.8), texreg (Version: 1.38.6), textshaping (Version: 0.3.6), TH.data (Version: 1.1-2), this.path (Version: 1.4.0), tibble (Version: 3.2.1), tidymodels (Version: 1.1.0), tidyquant (Version: 1.0.7), tidyr (Version: 1.3.0), tidyselect (Version: 1.2.0), tidyverse (Version: 2.0.0), tiff (Version: 0.1-11), timechange (Version: 0.2.0), timeDate (Version: 4022.108), timetk (Version: 2.8.3), tinytex (Version: 0.45), translations (Version: 4.2.2), tseries (Version: 0.10-54), tsfeatures (Version: 1.1), TTR (Version: 0.24.3), tune (Version: 1.1.1), TwoSampleMR (Version: 0.5.6), tzdb (Version: 0.3.0), urca (Version: 1.3-3), urlchecker (Version: 1.0.1), usethis (Version: 2.1.6), utf8 (Version: 1.2.3), uuid (Version: 1.1-0), vcd (Version: 1.4-11), vctrs (Version: 0.6.2), verification (Version: 1.42), VIM (Version: 6.2.2), vipor (Version: 0.4.5), viridis (Version: 0.6.3), viridisLite (Version: 0.4.2), vroom (Version: 1.6.3), waldo (Version: 0.5.0), warp (Version: 0.2.0), WeightIt (Version: 0.14.0), whisker (Version: 0.4.1), withr (Version: 2.5.0), workflows (Version: 1.1.3), workflowsets (Version: 1.0.1), xfun (Version: 0.39), xlsx (Version: 0.6.5), xlsxjars (Version: 0.6.1), XML (Version: 3.99-0.14), xml2 (Version: 1.3.4), xopen (Version: 1.0.0), xtable (Version: 1.8-4), xts (Version: 0.13.1), yaml (Version: 2.3.7), yardstick (Version: 1.2.0), zip (Version: 2.3.0), zoo (Version: 1.8-12)
